# Evolution of Pineal Nonvisual Opsins in Lizards and the Tuatara and Identification of Lepidopsin: A New Opsin Gene

**DOI:** 10.1093/gbe/evaf058

**Published:** 2025-05-02

**Authors:** Ricardo D Romero, Flávio S J de Souza

**Affiliations:** Instituto de Fisiología, Biología Molecular y Neurociencias (IFIBYNE-UBA-CONICET), Buenos Aires, Argentina; Departamento de Fisiología, Biología Molecular y Celular, Facultad de Ciencias Exactas y Naturales, Universidad de Buenos Aires (FBMC-FCEyN-UBA), Buenos Aires, Argentina; Instituto de Fisiología, Biología Molecular y Neurociencias (IFIBYNE-UBA-CONICET), Buenos Aires, Argentina; Departamento de Fisiología, Biología Molecular y Celular, Facultad de Ciencias Exactas y Naturales, Universidad de Buenos Aires (FBMC-FCEyN-UBA), Buenos Aires, Argentina

**Keywords:** gene duplication, photoreceptor, epiphysis, epithalamus, phototransduction

## Abstract

Many lizards (Squamata), as well as the tuatara (Rhynchocephalia), are distinguished among vertebrate groups for the presence of the parietal eye, or “third eye”, a structure derived from the pineal complex containing a simplified retina with photoreceptor cells. The parietal eye expresses nonvisual opsins that differ from the visual opsin repertoire of the lateral eyes. These are pinopsin (OPNP), parapinopsin (OPNPP), and parietopsin (OPNPT), all being evolutionary close to visual opsins. Here, we searched over 60 lepidosaurian genomes for pineal nonvisual opsins to check for the evolutionary trajectory of these genes in reptiles. Unexpectedly, we identified a novel opsin gene, which we termed “lepidopsin” (OPNLEP), that is present solely in the genomes of the tuatara and most lizard groups but absent from other vertebrates. Remnants of the gene are found in the coelacanth and some ray-finned fishes, implying that OPNLEP is an ancient opsin that has been repeatedly lost during vertebrate evolution. We found that the tuatara and most lizards of the Iguania, Anguimorpha, Scincoidea, and Lacertidae clades, which possess a parietal eye, harbor all pineal opsin genes. Lizards missing the parietal eye, like geckos, teiids, and a fossorial amphisbaenian, lack most or all pineal nonvisual opsins. In summary, our survey of pineal nonvisual opsins reveals (i) the persistence of a previously unknown ancient opsin gene—OPNLEP—in lepidosaurians; (ii) losses of nonvisual opsins in specific lizard clades; and (iii) a correlation between the presence of a parietal eye and the genomic repertoire of pineal nonvisual opsins.

## Graphical Abstract

**Graphical Abstract:**
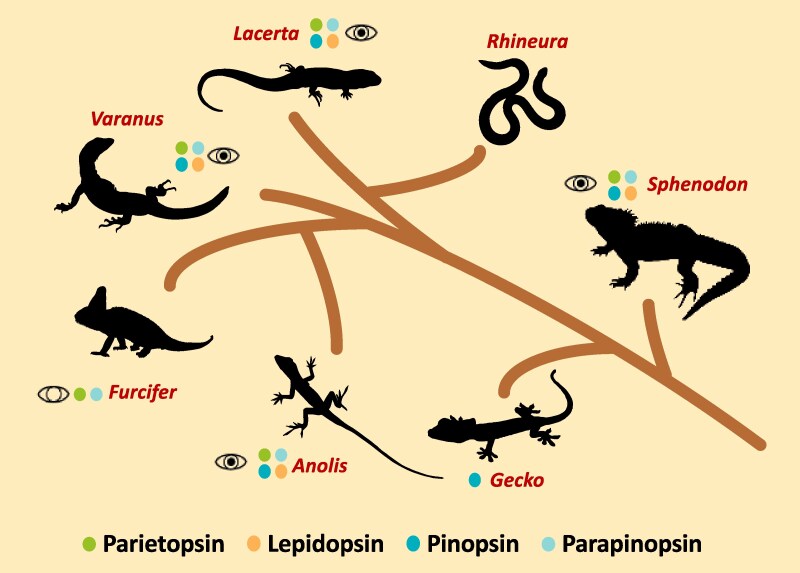

**Significance**

Many lizards have a unique structure located on the top of the head: a third eye, a sensory organ related to the pineal gland that was once common in vertebrates but that was lost from most lineages. This “eye” cannot form images but expresses specific light-absorbing opsin proteins. We analyzed the repertoire of pineal opsins in lizard genomes and observed that the presence of the third eye and pineal opsins are correlated. We also found a new opsin called lepidopsin that has been lost in all vertebrate lineages but has been retained in lizards and the tuatara, a kind of genetic “living fossil”. Our work illustrates how vertebrate evolution can proceed by the anatomical and genetic simplification of sensory organs.

## Introduction

In most animals, detecting light is an essential sensorial capability, employed to navigate and interact with the surrounding environment. In vertebrates, the best understood system of light detection is comprised by the two lateral eyes, which contain a layer of photoreceptor cells in the retina that absorbs photons and transmits visual information to the brain (Lamb 2013; Hagen et al. 2023). However, vertebrates can also detect light with other structures, like the pineal complex, a small group of sensory and endocrine brain organs derived from evaginations of the roof of the diencephalon during development. In mammals, the pineal complex is reduced to the pineal gland (epiphysis), which does not detect light by itself and serves a neuroendocrine function by secreting the hormone melatonin during nighttime. The pineal complex of other vertebrates, however, exhibits light sensitivity and may include other organs apart from the pineal proper (Oksche 1965; Dodt 1973; Eakin 1973; Vigh et al. 2002; Ekström and Meissl 2003; Mano and Fukada 2007). For instance, lampreys and teleost fishes have both a pineal and a parapineal organ with photoreceptor and neuroendocrine functions (Koyanagi et al. 2004, 2015). In anuran amphibians, the pineal complex comprises a deep pineal and a frontal organ (Stirnorgan), located under the skin between the eyes and capable of detecting light directly (Adler 1976; Korf et al. 1981). The frontal organ and the deep pineal emerge from the same primordium during anuran development (van de Kamer et al. 1962).

The pineal complex of the tuatara and many lizards comprises a deep neuroendocrine pineal and a parietal eye, located on the dorsal part of the head, often visible on a head scale as a parietal “spot” (Gundy and Wurst 1976a; Labra et al. 2010). The structure of the parietal eye resembles a lateral eye, with a lens and a simplified retina, being often nicknamed “third eye” for this reason, even though it cannot form images (Stebbins and Eakin 1958; Eakin 1973; Ung and Molteno 2004). In contrast to the anuran frontal organ, the evagination that gives rise to the reptilian parietal eye can be distinguished from the adjacent pineal evagination during development, indicating that it is likely homologous to the parapineal organ found in lampreys and teleosts (Oksche 1965; Eakin 1973; Meiniel 1977; Vigh et al. 2002). Anatomically, the parietal eye occupies an opening on the dorsal part of the skull—the pineal or parietal foramen—that allows exposure to light and the communication between the parietal eye and the brain through the parietal nerve (Engbretson et al. 1981). The presence of the parietal foramen in extant reptiles indicates the presence of a parietal eye and can be used to infer the presence of this sensory structure in extinct vertebrate lineages with ossified crania (Edinger 1955; Trost 1956; Benoit et al. 2016; Smith et al. 2018).

The retina of the parietal eye is lined with photoreceptor cells with a morphology similar to those of the lateral eyes, with a cilium-derived outer segment containing a dense arrangement of membranes (Eakin 1973; Solessio and Engbretson 1993). The capacity of detecting light depends on the presence of opsin proteins embedded in membrane stacks within the outer segments of the photoreceptors. Opsins are G protein-coupled transmembrane receptors responsible for detecting light. Their photosensitivity depends on a small chromophore moiety, like 11-*cis*-retinal, which is covalently bound to the opsin protein and can absorb photons. Light converts 11-*cis*-retinal to all-*trans*-retinal, triggering a conformational change of the opsin molecule and the activation of a G protein (Terakita 2005, 2012). The ancestral, basic repertoire of vertebrate visual opsins comprises five members, namely RHO/RH1, RH2, LSW, SWS1, and SWS2, each one with a particular light absorption profile. Vertebrate genomes, however, harbor many other opsin genes, which can be grouped into five families (OPN1, OPN3, OPN4, OPN5, and OPN6) following their phylogenetic relationships (Beaudry et al. 2017; Pérez et al. 2019).

Vertebrate visual opsins are included into the OPN1 group together with four so-called “nonvisual opsins”, namely (i) pinopsin (OPNP), (ii) parapinopsin (OPNPP), (iii) parietopsin (OPNPT), and (iv) vertebrate-ancient opsin (OPNVA). Many of these are expressed in photoreceptor cells of the pineal complex of nonmammalian vertebrates (Kawano-Yamashita et al. 2014; Pérez et al. 2019). OPNP has an absorption profile in the blue range (∼470 nm; Okano et al. 1994; Sato et al. 2018) and its expression has been detected in the pineal gland of several vertebrates like the chick, lamprey, the frog, *Xenopus laevis*, and some lizards (Okano et al. 1994; Max et al. 1995; Kawamura and Yokoyama 1997; Yokoyama and Zhang 1997; Frigato et al. 2006; Bertolesi et al. 2020) as well as the retina of a gecko lizard and several non-teleost fishes (Taniguchi et al. 2001; Sato et al. 2018). In the parietal eye of lizards, OPNP protein expression is found in the outer segments of photoreceptors of the iguanid *Uta stansburiana* together with OPNPT (Su et al. 2006). OPNPP has an absorption maximum in the ultraviolet range (∼370 nm; Koyanagi et al. 2004) and is expressed in the pineal complex of teleost fishes and lamprey (Blackshaw and Snyder 1997; Koyanagi et al. 2004, 2015), and the frog *X. laevis* (Bertolesi et al. 2020). In the parietal eye, OPNPP is coexpressed with OPNPT in photoreceptors of *Iguana iguana* (Wada et al. 2012). OPNPT, which absorbs light in the green range (∼522 nm; Su et al. 2006; Sakai et al. 2012), is expressed in the pineal complex of the zebrafish and *X. laevis* (Wada et al. 2012; Bertolesi et al. 2020). As mentioned, OPNPT protein is detected in the outer segments of photoreceptors of the parietal eye of iguanids, being coexpressed with OPNP in *U. stansburiana* and with OPNPP in *I. iguana* (Su et al. 2006; Wada et al. 2012). OPNVA, with a ∼500 nm absorption maximum (Sato et al. 2018), is mostly expressed in cells located in the deep brain of vertebrates and its expression in the pineal complex has not been detected, except in the pineal of the Atlantic salmon (Kojima et al. 2000; Philp et al. 2000; Bertolesi et al. 2020).

Although only a few species have been studied, previous work indicates that the parietal eyes of lizards express OPNP, OPNPP, and OPNPT. The photoreceptors of the parietal eye have a very unusual behavior in that they display chromatic antagonism: They depolarize in response to green light and hyperpolarize in response to blue light (Solessio and Engbretson 1993). Studies on *U. stansburiana* indicate that this antagonistic behavior is mediated by nonvisual opsins expressed together in the same photoreceptors (Su et al. 2006). Thus, the green light-responsive OPNP activates the G protein gustducin, leading to the activation of a cGMP-phosphodiesterase, a decrease in cGMP levels, the closure of cyclic nucleotide-gated channels (CNG), and the depolarization of the photoreceptor. The blue light-responsive OPNPT, on the other hand, activates Go, leading to the inhibition of a cGMP-phosphodiesterase, an increase in cGMP, the opening of CNGs, and the hyperpolarization of photoreceptor cells (Su et al. 2006; Kawano-Yamashita et al. 2014). Interestingly, the colocalization of OPNPP and OPNPT in photoreceptors of *I. iguana* indicates that a similar antagonistic chromatic response might exist between UV and green light detected by OPNPP and OPNPT, respectively (Wada et al. 2012). The photoreceptors synapse with ganglion cells located in the outer layer of the parietal eye retina, which project to the dorsolateral portion of the left habenula and other brain areas (Engbretson et al. 1981).

The functions of the parietal eye on lizard behavior and physiology have been studied in several species, usually by experiments in which the parietal eye is removed or covered to prevent it from detecting light (Tosini 1997). Lizards are environmental thermoregulators, and blocking parietal eye function alters the duration of exposure to sunlight and body temperature, as well as daily activity patterns (Stebbins and Eakin 1958; Hutchinson and Kosh 1974; Tosini 1997; Traeholt 1997). There is also evidence that the parietal eye is necessary for lizards to orient themselves in space (Ellis-Quinn and Simony 1991; Freake 2001) likely by using polarized sunlight as a compass (Beltrami et al. 2010). It may also be required for the control of metabolic rate and reproduction, possibly by regulating melatonin production by the pineal organ (Tosini 1997). In spite of the important functions associated with it, the parietal eye is not present in all lizards. Gundy and Wurst (1976a) found that 12 out of 18 families of “Larcetilia” (excluding amphisbaenians and snakes) had parietal eyes, with all members of some important families, like Gekkota, Dibamidae, and Teiidae lacking parietal eyes altogether. In addition, snakes, which are phylogenetically grouped with Anguimorpha and Iguania in the Toxicofera clade, also lack parietal eyes.

Even though nonvisual OPN1 opsins are ancient genes, previous studies have found that these genes have a somewhat patchy distribution in vertebrate groups. In particular, *OPNP*, *OPNPP*, and *OPNPT*, which are expressed in the lizard parietal eye, have frequently been lost during evolution. Thus, snakes have lost *OPNP*, *OPNPP*, and *OPNPT*, turtles have lost *OPNPP* and *OPNPT*, and crocodylians, which completely lack a pineal complex, lost *OPNP*, *OPNPP*, and *OPNPT* (Emerling 2017a, 2017b). Birds lack *OPNPP* and *OPNPT*, while mammals are unique in having lost all nonvisual OPN1 opsins (Hankins et al. 2014; Emerling 2017a). In contrast, the genome of frogs *X. laevis* and *Xenopus tropicalis*, which have a frontal organ similar to the parietal eye, contains all nonvisual OPN1 opsins (Bertolesi et al. 2020). The presence of these opsins seems to be linked to the occurrence of photosensitive organs of the pineal complex of vertebrates, like the anuran frontal organ and the reptilian parietal eye. Indeed, *OPNP*, *OPNPP*, and *OPNPT* are all expressed in the developing pineal complex of *X. laevis* (Bertolesi et al. 2020).

Lizards and snakes comprise the Squamata, with over 11,000 species (including over 7,600 lizards) displaying a high diversity of form and behavior (Meiri 2024). Together with Rhynchocephalia, of which the tuatara of New Zealand (*Sphenodon punctatus*) is the sole extant representative, Squamata is included within Lepidosauria. It is estimated that Rhynchocephalia and Squamata diverged in the late Permian, before 250 million years ago (MYA), while extant squamate groups diversified during the Jurassic and later (after 200 MYA, Simões et al. 2020, 2022). To extend the analyses of pineal photosensitivity and opsin evolution to this understudied group of animals, we sought in this work to identify the repertoire of genes encoding nonvisual OPN1 opsins in the genomes of Lepidosauria. In this process, we discovered a new nonvisual OPN1 opsin, *lepidopsin* (*OPNLEP*), which is phylogenetically similar to *OPNPP*. In lizard groups, we found that the presence of a parietal eye is associated with the presence of *OPNPP*, *OPNPT*, and *OPNLEP*, while *OPNP* is frequently lost. Fragments of *OPNLEP* exons are seen in the genomes of snakes, turtles and crocodylians, showing that this gene was once functional in all stem reptilians. Surprisingly, fragments of the *OPNLEP* gene are present in the genome of the coelacanth and some basal groups of ray-finned fishes, implying that the gene was repeatedly lost during evolution, likely due to the loss of the parietal eye in most sarcopterygian and tetrapod clades.

## Results

### Discovery of a New Opsin: OPNLEP

**Fig. 1.**
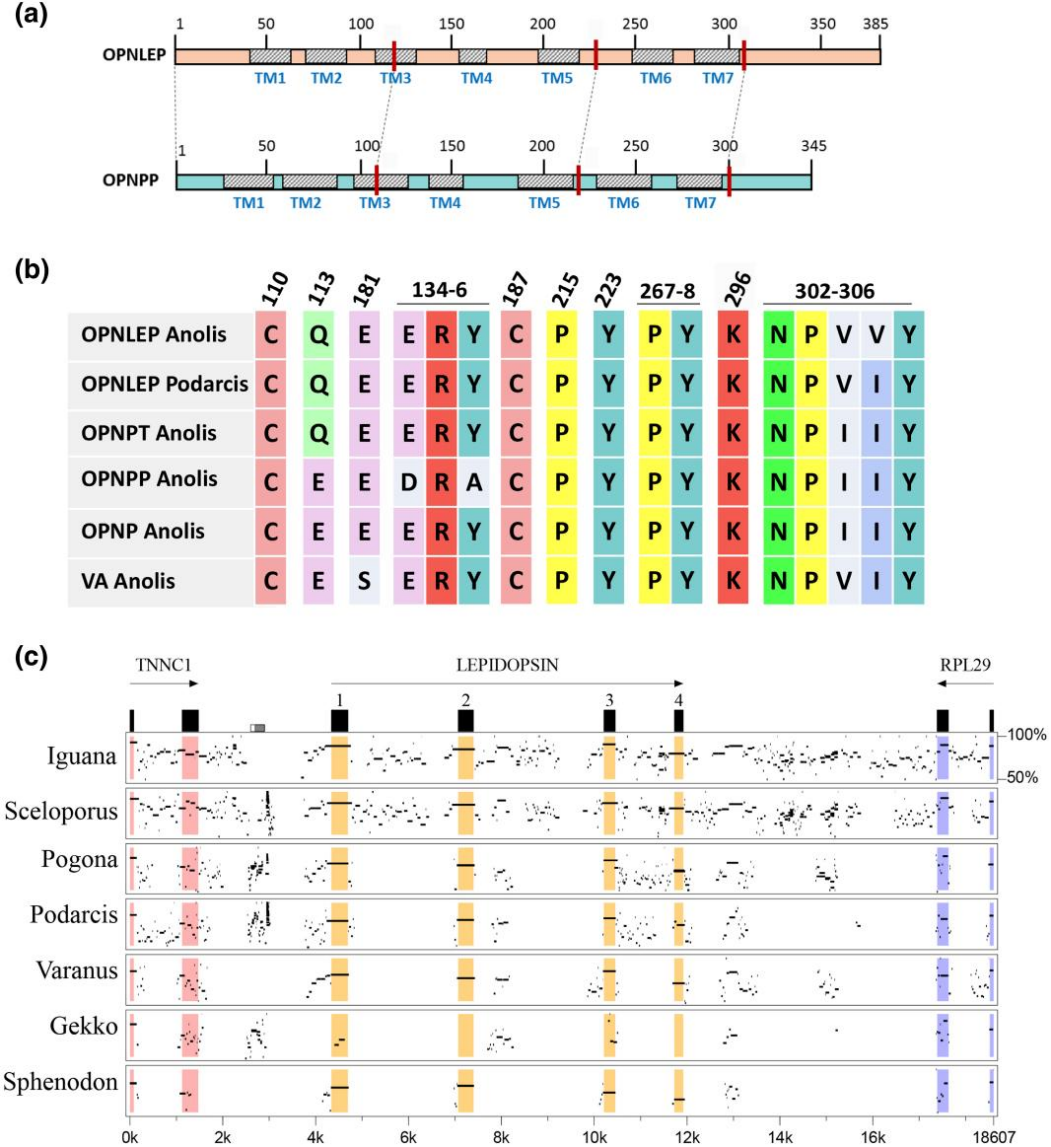
Characterisation of a new reptilian opsin, lepidopsin. a) Schematics of the structure of OPNLEP and OPNPP proteins of the green anole, *A. carolinensis*. TM: transmembrane domains 1-7. Red bars and dashed lines point to the approximate positions of the three introns in both sequences. b) Key residues involved in opsin function in five nonvisual OPN1 opsins from the green anole and the OPNLEP of the common wall lizard (*Podarcis muralis*). Residue numbers correspond to bovine rhodopsin. c) Percentage identity plot (PipMaker program) between the nucleotide sequence of the green anole OPNLEP locus and homologous loci from other lepidosaurian species: *Iguana delicatissima*, *Sceloporus undulatus*, *P. muralis*, *Varanus komodoensis*, *Gekko japonicum* and *S. punctatus*. The last two exons of *TNNC1* and *RPL29* are also shown in the plot.

A search for nonvisual opsins of the OPN1 group in lizard genomes readly reveals the presence of all four known genes, namely *OPNP*, *OPNPP*, *OPNPT*, and *OPNVA* (see below). Unexpectedly, we found a fifth nonvisual OPN1 opsin gene in the tuatara and in most lizard genomes, which we named *Lepidopsin* (*OPNLEP*) for its occurrence in Lepidosauria. The predicted amino acid sequence of OPNLEP proteins ranges from 376 to 385 residues, and it aligns well with that of other opsins, including the seven transmembrane (TM) domains (Fig. 1a and supplementary fig. S1, Supplementary Material online). It has all the typical conserved residues found in opsins, like cysteines 110 and 187, that form a disulfide bond, and lysine 296 in TM7, which binds the retinal moiety necessary for light absorption (Fig. 1b, residues are numbered according to bovine rhodopsin). Interestingly, glutamate 113, which serves as counterion to the protonated Schiff base of the retinal chromophore in most OPN1 opsins, is changed to glutamine in OPNLEP, as in OPNPT (Su et al. 2006; Fig. 1b). Thus, it is probable that the Schiff base counterion in OPNLEP is glutamate 181, as has been shown for OPNPT (Sakai et al. 2012).

In all lepidosaurians analyzed, the *OPNLEP* gene is flanked by *TNNC1* (encoding troponin c1) and *RPL29* (encoding ribosomal protein L29; Fig. 1c and supplementary fig. S2, Supplementary Material online). A global nucleotide sequence alignment analysis with MultiPipMaker (Schwartz et al. 2000) using as reference the *OPNLEP* locus of the green anole (*Anolis carolinensis*) shows that the four exons of the gene are highly conserved in lepidosaurians (Fig. 1c). The exons of the green anole are particularly evident in the alignment with the tuatara locus, confirming that *OPNLEP* has four coding exons. This is the same number of exons as *OPNPP* and *OPNPT*, while *OPNP* and *OPNVA* genes have five. In addition, the four predicted exons of *OPNLEP* correspond to the same, homologous exons of *OPNPP* and *OPNPT* (Fig. 1a). Interestingly, in species with chromosome-level assemblies, we found that *OPNLEP* and *OPNPP* are located in the same chromosomes separated by similar distances, while other nonvisual OPN1 genes are found in different chromosomes (supplementary fig. S2, Supplementary Material online; supplementary table S1, Supplementary Material online). This suggests that *OPNLEP* and *OPNPP* genes may have originated by a tandem or segmental duplication. A further analysis of the origin of *OPNLEP* is given in a section below.

### Survey of Nonvisual OPN1 Opsins in Lepidosauria

We took advantage of the availability of the tuatara and many lizard genomes to search for nonvisual opsins of the OPN1 group, including OPNLEP, using the BLAST program. The identity of the genes was determined by phylogenetic analysis (see below) and by the genomic neighborhood of the genes, since we observed that the synteny of opsin genes is highly conserved in lepidosaurs (supplementary fig. S2, Supplementary Material online; see Materials and Methods). As a general evolutionary framework, we followed recent molecular phylogenetic analyses of squamates (Pyron et al. 2013; Burbrink et al. 2020; Simões and Pyron 2021). Our survey includes representatives of Rhynchocephalia (the tuatara) and most large clades of lizards, namely Gekkota (9 species from 5 families), Iguania lizards belonging to Pleurodonta (11 species from 5 families) and Acrodonta (12 species from 2 families), Lacertoidea (16 species from 4 families), Anguimorpha (6 species from 5 families), and Scincoidea (7 species from 2 families). Although included in Squamata, we have not analyzed snake (Serpentes) genomes as previous analyses have already shown the loss of *OPNP*, *OPNPP*, and *OPNPT* genes from this clade (Emerling 2017b), and we could not find a complete *OPNLEP* gene in snakes (see below).

OPNP, OPNPP, and OPNPP are all known to be expressed in the parietal eye of lizards (Kawamura and Yokoyama 1997; Taniguchi et al. 2001; Su et al. 2006; Wada et al. 2012), but this structure is not present in all groups. To check for a correlation between opsin repertoire and the presence of the parietal eye, we considered the occurrence of a parietal eye or a parietal “spot” from data gathered by Gundy and Wurst (1976a, 1976b) and Lee (1998), as well as by the presence of a pineal opening or parietal foramen in the skull, which generally indicates the presence of a parietal eye (Edinger 1955; Trost 1956; see Materials and Methods).

**Fig. 2.**
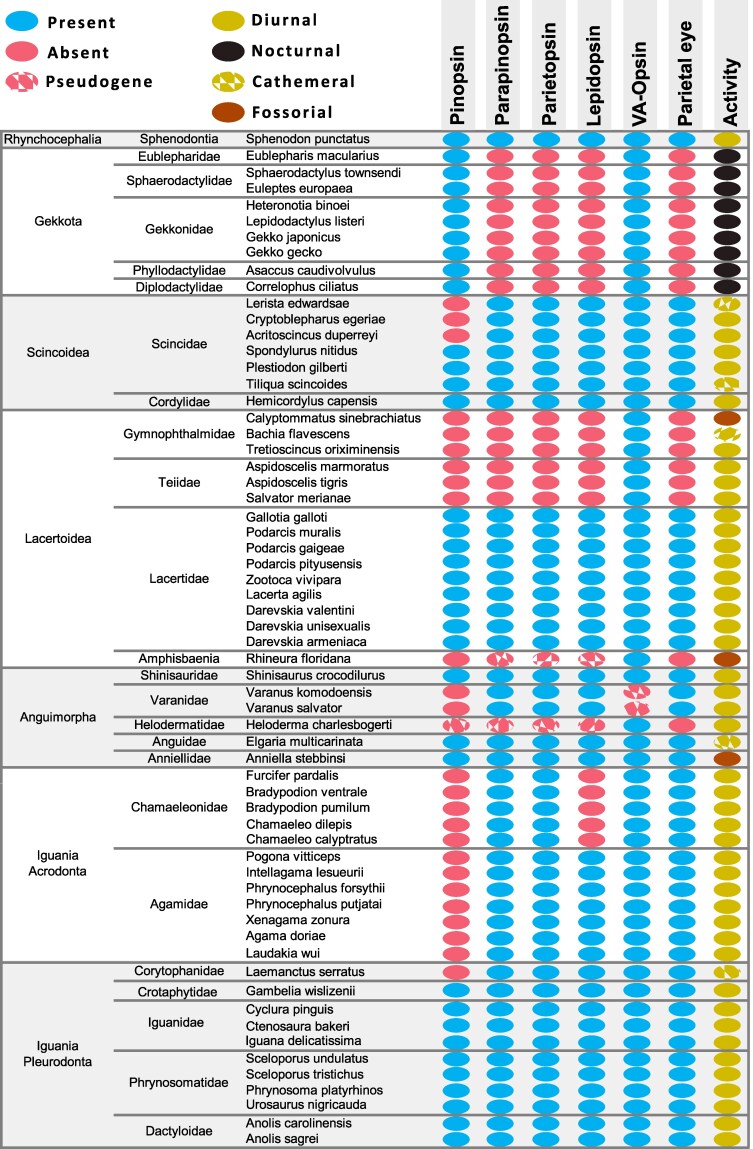
Distribution of nonvisual OPN1 opsins among lepidosaurians. The presence of a parietal eye, as well as patterns of activity or fossoriality, are indicated.

The distribution of the OPN1 nonvisual opsins in 63 lepidosaurian genomes is shown in Fig. 2. Our observations can be summarized as follows:

Rhynchocephalia: The tuatara genome has all opsins surveyed, including *OPNLEP*. The tuatara has a well-developed parietal eye, with a retina and lens, placed inside a large parietal foramen (Dendy 1911; Edinger 1955; Ung and Molteno 2004).
Gekkota: Gecko genomes have intact *OPNP* and *OPNVA* genes, but all species surveyed have lost the other three opsin genes. Geckos are known to have lost the parietal eye (Gundy and Wurst 1976a, 1976b). In contrast to other lizards, most gecko species are nocturnal and may have experienced a “nocturnal bottleneck” during their early evolution (Katti et al. 2019; Pinto et al. 2019; Kojima et al. 2021).
Scincoidea: The African girdle lizard *Hemicordylus capensis* (Cordylidae) has all opsins surveyed. As for skinks (Scincidea), all have all opsins except for *OPNP*, which is lost in three of the species we analyzed. Both cordylids and skinks have parietal eyes (Gundy and Wurst 1976b).
Lacertoidea: Lizards of the Lacertidae family have all five opsins and well-developed parietal eyes (Trost 1956; Gundy and Wurst 1976a, 1976b). The opsin repertoire and status of the parietal eye in other families are different. Teiids and gymnophthalmids are related groups (Goicoechea et al. 2016) that lack a parietal eye (Gundy and Wurst 1976b; Lee 1998). Interestingly, the six species surveyed lack all opsins except for *OPNVA*. For amphisbaenians, which lack a parietal eye and are of fossorial habits (Lee 1998; Gans and Montero 2008), we analyzed the *Rhineura floridana* genome (Florida worm lizard). *Rhineura floridana* has recognizable *OPNPP*, *OPNPT*, and *OPNLEP* genes that nevertheless carry mutations that should render them pseudogenes (supplementary table S2, Supplementary Material online). It lacks *OPNP*, and its only nonvisual OPN1 gene that seems to be functional is *OPNVA*.
Anguimorpha: Monitor lizards of the Varanidae family have a well-developed parietal eye, and the two species that we surveyed (*Varanus komodoensis* and *Varanus salvator*) have *OPNPP*, *OPNPT*, and *OPNLEP*, while *OPNP* is lost and the *OPNVA* gene lacks its first and fifth exons and is thus pseudogenized. The representatives of Anguidae and Shinisauridae families, on the other hand, have all five opsin genes and also possess a parietal eye. *Anniella stebbinsi* also has all opsin genes surveyed, even though it belongs to a small group of Californian legless lizards of fossorial habit (Anniellidae; Papenfuss and Parham 2013). Interestingly, *Anniella pulchra*, a related species, lacks a parietal foramen but still has a well-formed parietal eye located under the skull (Gundy and Wurst 1976a). Finally, the Guatemalan beaded lizard (*Heloderma charlesbogerti*), belonging to a family (Helodermatidae) lacking parietal eyes, has all nonvisual OPN1 opsins in a pseudogenized state except for *OPNVA*.
Pleurodont iguanians: Pleurodonts have all five opsins, although one species, *Laemanctus serratus*, has a pseudogenized *OPNP*. Iguanians have a well-developed parietal eye (Trost 1956; Gundy and Wurst 1976a, 1976b), and indeed the expression of *OPNP*, *OPNPT*, and *OPNPP* expression has been studied in detail in the pineal eye photoreceptors of two pleurodont species, namely *U. stansburiana* and *I. iguana* (Su et al. 2006; Wada et al. 2012).
Acrodont iguanians: Both agamids and chamaeleonids lack *OPNP*, while chamaleonids lack, in addition, *OPNLEP*. Both groups, however, have parietal eyes (Trost 1956; Gundy and Wurst 1976a, 1976b). Interestingly though, chamaeleonids of the *Chameleo* genus have a somewhat degenerate parietal eye, described as a hollow vesicle lacking lens and retina (Edinger 1955; Gundy and Wurst 1976a).

In general, we observe that genomes from lepidosaur species that have parietal eyes possess *OPNPP*, *OPNPT*, and *OPNLEP*, while species and groups where this structure is missing (geckos, teiids, gymnophthalmids, amphisbaenians, helodermatids) lack all three functional genes.

### Evolutionary Origin of OPNLEP

**Fig. 3.**
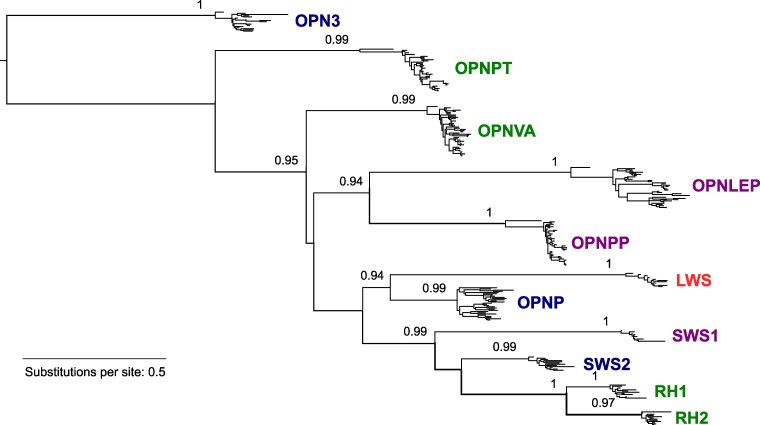
OPNLEP proteins comprise a new nonvisual opsin clade. Phylogenetic tree of nonvisual and visual OPN1 opsin proteins of lepidosaurians estimated using the maximum likelihood method (PhyML 3.0). Opsins of the OPN3 group were included to root the tree. Label colours indicate approximate absorption maximum of opsins (Hankins et al. 2014; Hagen et al. 2023; Gyoja et al. 2025). Individual species and proteins are not shown for clarity. Support values >0.9 (SH-like aLRT test) of main clades are indicated. A detailed tree with species names is found in supplementary fig. S3, Supplementary Material online.

A complete gene for the new nonvisual OPN1 opsin, *OPNLEP*, is found in 40 out of 63 lepidosaurian species, and 14 out of 24 lepidosaurian families, that we analyzed (Fig. 2). To confirm that *OPNLEP* is a new OPN1 gene, we analyzed the phylogenetic relationships between all groups of lepidosaurian OPN1 genes, both nonvisual (OPNP, OPNPP, OPNPT, and OPNVA) and visual (LWS, SWS1, SWS2, RH1, and RH2). Opsins of the OPN3 (encephalopsin) clade, which are expressed in the brain (Blackshaw and Snyder 1999; Pérez et al. 2019) and have previously been found to be the closest to OPN1 opsins (Beaudry et al. 2017), were used as a root in the analysis. The resulting maximum likelihood tree (Fig. 3 and supplementary fig. S3, Supplementary Material online) shows OPNLEP sequences grouped in a well-defined branch with high statistical support, indicating that it constitutes a distinct OPN1 opsin clade. OPNP clusters with visual opsins, as observed in previous studies (Beaudry et al. 2017; Bertolesi et al. 2020), while OPNLEP occupies a well-supported branch together with OPNPP. A close-up of the OPNLEP branch shows the expected topology, with the tuatara sequence at the base of the clade and the other branches corresponding to the lizard taxonomic groups analyzed (supplementary fig. S4, Supplementary Material online).

Many species of lizards have a *OPNLEP* gene that carries several mutations that render them pseudogenes, as is the case of the amphisbaenian *R. floridana* and the beaded lizard, *H. charlesbogerti* (supplementary table S2, Supplementary Material online). In geckos, fragments of the *OPNLEP* gene are still present, but not the whole gene. For instance, the nucleotide global alignment of Fig. 1c shows that fragments of exons 1 and 3 of *OPNLEP* are still detectable in *Gekko japonicus*, while other exons are absent.

**Fig. 4.**
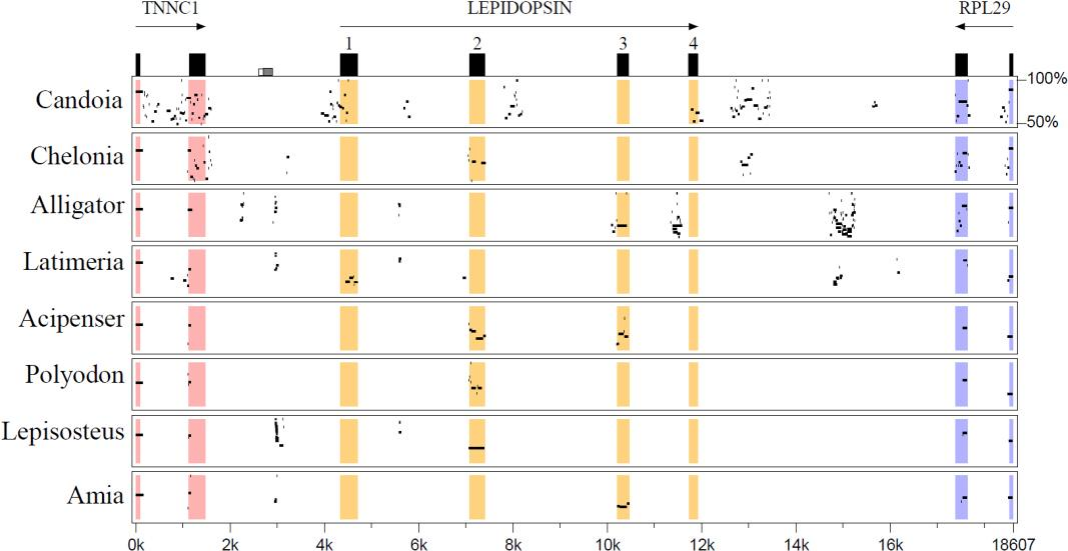
Remnants of OPNLEP exons are still found in several vertebrate clades. Percentage Identity Plot (MultiPipMaker) of the green anole OPNLEP locus and the homologous loci of the Papuan ground boa (*Candoia aspera*), green sea turtle (*Chelonia mydas*), American alligator (*Alligator mississipiensis*) and coelacanth (*Latimeria chalumnae*). Ray-finned fishes represented are the sterlet (*Aciperser ruthenus*), paddlefish (*Polyodon spathula*), spotted gar (*Lepisosteus oculatus*) and bowfin (*Amia calva*). The two last exons of both flanking genes, *TNNC1* and *RPL29*, are present in all species.

Although BLAST searches indicate that a complete *OPNLEP* gene is absent from other reptiles, we used MultiPipMaker to check for remnants of the gene between *TNNC1* and *RPL29* in the genomes of other reptile clades. We found that fragments of *OPNLEP* exons are recognizable in snakes like *Candoia aspera* and *Thamnophis elegans*, turtles like *Chelonia mydas* and *Gopherus evgoodei*, and crocodilians like *Alligator mississippiensis*, indicating that the origin of *OPNLEP* predates lepidosaurians (Fig. 4 and supplementary table S3, Supplementary Material online).

When we extended the search to other lobe-finned fishes (Sarcopterygii), we were unable to identify *OPNLEP* remnants in amphibians, birds, and mammals, as well as lungfishes (supplementary fig. S3, Supplementary Material online). Remarkably, most of the exon 1 of *OPNLEP* is readily recognizable in the genome of the coelacanth, *Latimeria chalumnae*, between the homologs of *TNNC1* and *RPL29* (Fig. 4). Among ray-finned fishes (Actinopterygii), long remnants of *OPNLEP* exons were detectable in the genomes of members of the Holostei clade, namely the spotted and longnose gars (*Lepisosteus oculatus* and *Lepisosteus osseus*) and the bowfin (*Amia calva*), as well as Acipenseriformes like the sturgeon (*Acipenser ruthenus*) and paddlefish (*Polyodon spathula*), in all cases between the *TNNC1* and *RPL29* genes (Fig. 4 and supplementary table S3, Supplementary Material online). *OPNLEP* was not retrieved from the genomes of bichirs (Polypteriformes) and the teleosts, yellowfin tuna (*Thunnus albacares*), and zebrafish (*Danio rerio*). Among other vertebrate groups, we were unable to detect *OPNLEP* remnants in cartilaginous (Chondrichthyes) or jawless (Cyclostomata) fishes (supplementary table S3, Supplementary Material online). These results indicate that *OPNLEP* is an ancient opsin gene that originated before the divergence of lobe- and ray-finned fishes but has been retained only in recent lepidosaurians.

## Discussion

In this work, we describe the repertoire of nonvisual opsins of the OPN1 group in available lepidosaurian genomes. Our main observations are that (i) lepidosaurians have a new OPN1 gene, *OPNLEP*, most similar to *OPNPP*; (ii) the repertoire of nonvisual opsins varies among lizards, with a tendency for lizard taxa lacking the parietal eye also lacking *OPNPP*, *OPNPT*, and *OPNLEP* genes; and (iii) *OPNLEP* is an ancient gene, having originated at least in the beginning of the evolution of bony fishes (Osteichthyes).

In a phylogenetic tree, OPNLEP proteins appear closer to OPNPP than to other OPN1 opsins. The three genes have four exons and three introns with splicing sites located in the same relative positions, indicating an origin by duplication from an ancestral gene. In addition, *OPNLEP* and *OPNPP* genes are always linked in the same chromosome of lizards. This observation aligns with the scenario proposed by Lagman et al. (2024), in which several rounds of tandem duplications gave origin to OPN1 opsin genes in ancient vertebrates. In fact, the gene referred by them as “parapinopsin-like” in the spotted gar genome (Lagman et al. 2024) might correspond to the *OPNLEP* fragment in this species. The amino acid sequence of OPNLEP resembles OPNPT in having a glutamine at position 113, instead of glutamate, so it is likely that the conserved glutamate 181 in OPNLEP is the counterion to the Schiff base linkage between the retinal chromophore and lysine 296, as is the case for OPNPT and invertebrate opsins (Sakai et al. 2012).

**Fig. 5.**
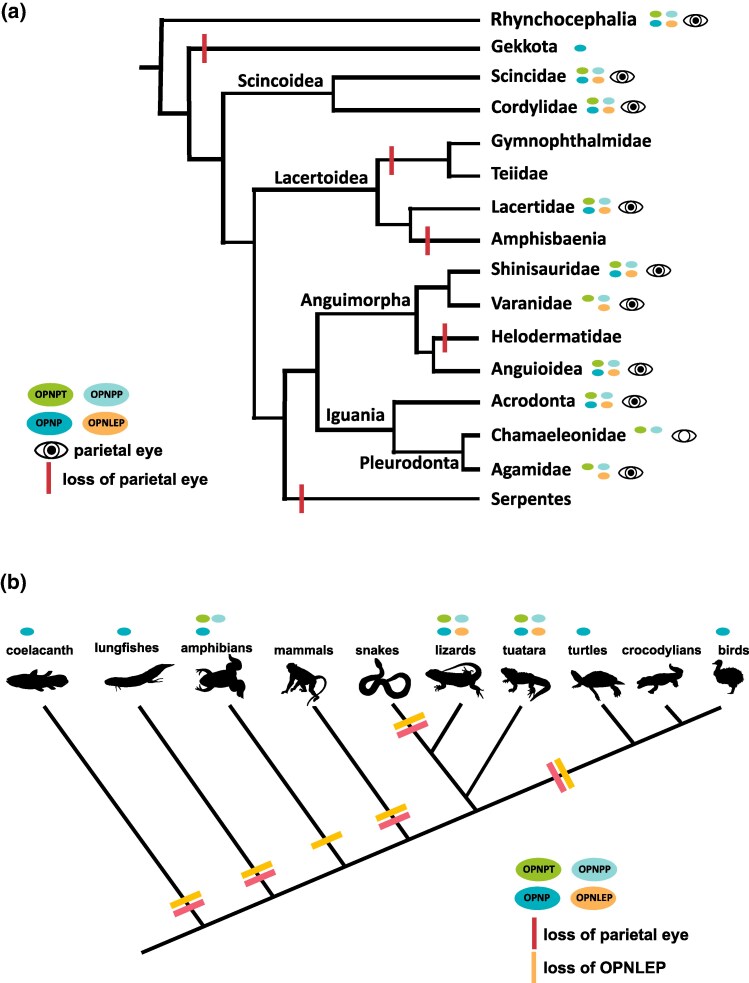
Evolution of OPN1 nonvisual opsins and parietal eye in vertebrates. a) Phylogenetic relationships between lepidosaurian clades showing the correlation between the presence of a parietal eye and pineal nonvisual opsins. OPNPP, OPNPT and OPNLEP tend to be present in clades with well-developed parietal eyes. b) Sarcoptergygian phylogenetic relationships and their repertoire of OPN1 nonvisual opsins. The parietal eye and OPNLEP have been independently lost several times during evolution.

Our survey of nonvisual OPN1 opsins in lepidosaurians revealed some important tendencies in the evolution of these genes, summarized in Fig. 5a. The tuatara has maintained the ancestral repertoire of five nonvisual opsins. In lizards, *OPNVA* seems to be the least likely to be lost, missing only from varanids. *OPNVA* is expressed in neurons located in the deep brain, presumably rendering it less dependent on the function of the eyes and the pineal complex. It is also present in snakes (Emerling 2017b). *OPNP*, on the other hand, was lost in several clades, including all agamids, several skinks, varanids, teiids, and gymnophthalmids, as well as the beaded lizard and the amphisbaenian, *R. floridana*. It is expressed in the retina and parietal eyes of lizards (Taniguchi et al. 2001; Su et al. 2006; Sato et al. 2018). *OPNPP*, *OPNPT*, and *OPNLEP* are present in lizards exhibiting a parietal eye and missing in clades where this sensory structure is not present. Thus, the three genes have been lost in geckos, teiids, gymnophthalmids, the beaded lizard, and the fossorial amphisbaenian *R. floridana*, all of which lack parietal eyes. The only exception is chamaleonids, which have lost *OPNLEP* but maintained the other two opsins. The parietal eye of chameleons, at least those of the *Chameleo* genus, has a degenerated structure (“a hollow vesicle”, Gundy and Wurst 1976a), and it may not be as functional as in other agamids. Thus, in general, the conservation of *OPNPP*, *OPNPT*, and *OPNLEP* in lepidosaurian genomes seems to depend on the presence of the parietal eye. Interestingly, this correlation is also observed in *A. stebbinsi*, which although being from a fossorial lizard family (Anniellidae) that lacks a parietal foramen, has a well-developed parietal eye under the skull (Gundy and Wurst 1976a, 1976b) and intact *OPNPP*, *OPNPT*, and *OPNLEP* genes.

In general terms, the fact that lizards with parietal eyes tend to have a complete repertoire of nonvisual OPN1 opsins is consistent with the observation that *OPNP*, *OPNPP*, and *OPNPT* expression has been observed in photoreceptor cells of the lizard parietal eye (Su et al. 2006; Wada et al. 2012). *OPNP*, *OPNPP*, and *OPNPT* are also expressed in the pineal complex of the frog *X. laevis* (Bertolesi et al. 2020), which has a frontal organ similar to a parietal eye (van de Kamer et al. 1962; Eakin 1973). Thus, we consider very likely that future studies will reveal that *OPNLEP* is also expressed in the parietal eyes of lepidosaurians.

From an evolutionary and ecological standpoint, the profile of opsin losses in lepidosaurians generally agrees with the idea that nocturnality and fossoriality favor the reduction or loss of photosensitive organs, like the parietal eye, and associated genes. Snakes, which belong to the clade Toxicofera together with Iguania and Anguimorpha, seem to have evolved from nocturnal and/or fossorial ancestors (Hsiang et al. 2015), influencing adaptations like the loss of limbs, structural changes in retinal photoreceptors and loss of two visual opsins (*SWS2* and *RH2*; Simões et al. 2015; Emerling 2017b; Katti et al. 2019). Snakes have lost the parietal eye as well as *OPNP*, *OPNPP*, and *OPNPT*, and only remnants of the *OPNLEP* gene are still detectable. The gecko lineage also seems to have undergone a “nocturnal bottleneck” during evolution, when their eyes adapted to reduced light conditions and many genes were lost, including two visual opsins (*SWS2* and *RH1*; Emerling 2017b; Pinto et al. 2019; Katti et al. 2019; Kojima et al. 2021). Emerling (2017b) showed that *G. japonicus* lost *OPNPP* and *OPNPT*, and we have now extended this observation to *OPNLEP* and eight additional species from five different families, which suggests that the nocturnal lifestyle led to the loss of the parietal eye and these three opsins from the gecko lineage. Amphisbaenians are legless fossorial lizards with thick scales, reduced eyes, and missing parietal eyes. Consistent with this, *R. floridana* has lost *OPNP* and pseudogenized *OPNPP*, *OPNPT*, and *OPNLEP*. It remains to be seen if the loss of these opsins will be a general feature of amphisbaenians. Finally, extant archelosaurians (turtles, crocodilians, and birds) also lack parietal eyes and lost *OPNPP*, *OPNPT* (Emerling 2017a), and only have remnants of *OPNLEP*.

Remarkably, even though *ONPLEP* could not be retrieved from the genomes of amphibians, birds, mammals, and other vertebrates, we did identify exon remnants in the coelacanth and in two groups of ray-finned fishes, Acipenseriformes and Holostei. In all these species, the *OPNLEP* fragments are located in the same, homologous locus where the gene is found in lepidosaurians. The slow pace of molecular and morphological evolution of coelacanths (Amemiya et al. 2013; Cavin and Guinot 2014; Toriño et al. 2021; Clement et al. 2024), sturgeons, the paddlefish, gars, and bowfins (Redmond et al. 2023; Brownstein et al. 2024) may account for this survival. Thus, even though complete *OPNLEP* genes are only present in lepidosaurs, this opsin is an ancient one, dating from before the divergence between ray- and lobe-finned fishes, in the middle Silurian, around 430 million years ago (MYA; Brazeau and Friedman 2015). Among sarcopterygians, this implies that *OPNLEP* was independently pseudogenized and lost in the lineages leading to extant coelacanths, lungfishes, amphibians, mammals, and archelosaurians (turtles, crocodiles, and birds; Fig. 5b). Interestingly, a pineal or parietal eye is a primitive feature of bony fishes, since the parietal foramen, where the parietal eye sits, is an ancestral character (plesiomorphy) present in the fossilized crania of ancient fishes belonging to the ray- and lobe-finned lineages (Teng et al. 2019). Thus, parietal foramina are found in late Silurian bony fish like *Guiyu* and *Psarolepis* (Yu 1998; Zhu et al. 1999, 2009), early Devonian sarcopterygian fish like *Ligulalepis* and *Styloichthys* (Zhu and Yu 2002; Clement et al. 2018), Devonian actinopterygians like *Meemannia*, *Mimipiscis*, and *Raynerius* (Zhu et al. 2006; Choo 2011; Giles et al. 2015; Lu et al. 2016) as well as late Devonian tetrapodomorphs like *Ventastega*, *Tiktaalik*, *Acanthostega*, and *Ichtyostega* (Daeschler et al. 2006; Ahlberg et al. 2008). Much later, during the Triassic and Jurassic, tetrapod lineages started to lose parietal foramina from the center of the skull, as has been documented in detail for the therapsid lineage leading to the first mammals (Benoit et al. 2016). Thus, early sarcopterygians and tetrapods had both a parietal eye and *OPNLEP*, and it is tempting to speculate that, as the parietal eye was lost in later lineages, the *OPNLEP* gene faced the same fate (Fig. 5b).

As this manuscript was under review, a paper by Gyoja et al. (2025) appeared that describes the independent discovery of OPNLEP in the genome of a few reptiles, which the authors named Q113-bistable opsin. Interestingly, *A. carolinensis* OPNLEP/QB is a bistable opsin that absorbs light in the UV range, with a peak at 360 nm (Gyoja et al. 2025). Thus, the photochemical characteristics of OPNLEP seem to be similar to that of OPNPP.

In conclusion, we have outlined the evolution of nonvisual OPN1 opsins in lepidosaurians and discovered a new opsin gene in this group. Our results imply a functional relation between the genomic repertoire of opsin genes and the parietal eye and habits of lizards, and as more genomic information accumulates and more species are represented, the details of nonvisual opsin evolution in this highly diverse clade will become clearer. As for OPNLEP, future studies are needed to address its expression sites and physiological roles, in particular in relation to the reptilian third eye.

## Materials and Methods

### Genomic Database Searches

We used databases containing complete genome sequences of squamates and the tuatara, namely Ensembl (http://ensembl.org/index.html/), Ensembl Rapid Release (https://rapid.ensembl.org/index.html/), and, specially, the NCBI Genome Database (https://www.ncbi.nlm.nih.gov/datasets/genome/) (supplementary table S4, Supplementary Material online). Initially, the well-characterized predicted protein sequences of OPNP, OPNPP, OPNPT, and OPNVA from *X. laevis* and *X. tropicalis* (Bertolesi et al. 2020) were used to find the corresponding genes in *A. carolinensis* and a few other lizards using the TBLASTN searching tool. Often, the lizard genes had been automatically annotated with misleading names (for instance “parapinopsin-like” for *OPNVA*). Then, the protein sequences from lizards were used to systematically search the genomes lepidosaurians for orthologue genes with TBLASTN. Alignments of the protein sequences with CLUSTAL OMEGA (Madeira et al. 2024) were used to confirm the identity of each opsin gene. We found that the genomic context (microsynteny) around the opsin genes was conserved, a feature that also helped identify the genes (supplementary fig. S2, Supplementary Material online). In the process of collecting the protein sequences of the nonvisual OPN1 genes, we identified a new member of the group, *Lepidopsin* (*OPNLEP*), and repeated the analysis with this gene. The OPNLEP protein sequences analyzed are compiled in supplementary table S5, Supplementary Material online.

### Assessing the Functionality of Opsin Genes

The integrity and functionality of the four previously known nonvisual opsin genes were evaluated by verifying that all exons could be retrieved from the genomes (four exons for *OPNPP* and *OPNPT*, five exons for *OPNP* and *OPNVA*) and that the whole protein sequence was predicted without frameshifts. For *OPNLEP*, which is a new gene without a standard sequence, we analyzed in detail the *OPNLEP* loci from diverse lepidosaurians, namely *S. punctatus*, *A. carolinensis*, *Pogona vitticeps*, *Podarcis muralis*, *V. komodoensis*, and *H. capensis*, to identify the precise boundaries of the four exons with their splicing sites and the coding sequence of the mRNAs.

To help in identifying exons, we used the global nucleotide alignment program MultiPipMaker (Schwartz et al. 2000; http://pipmaker.bx.psu.edu/pipmaker/). A 43 kb region encompassing the *OPNLEP* gene of *A. carolinensis*, including neighboring genes *TNNC1* and *RPL29*, was used as default in MultiPipMaker to be compared to the homologous loci of other species and identify coding exons and splice sites. When the predicted protein sequences of opsins could not align smoothly with well-characterized proteins, as in the case of *OPNLEP* of *R. floridana* and *H. charlesbogerti*, we performed MultiPipMaker analyses with nucleotide sequences and visually inspected the alignments to identity indels, frameshifts, and mutations in splice sites, so as to confirm their categorization as pseudogenes. Similar analyses were performed with *OPNP*, *OPNPP*, and *OPNPT*. The probable pseudogenes identified and the mutations that they carry are listed in supplementary table S3, Supplementary Material online. MultiPipMaker analyses also allowed for the identification of remnants of *OPNLEP* exons, as in geckos, snakes, and other reptiles and vertebrates.

### Morphological and Activity Data

The presence of a pineal/parietal eye in lizards of particular species, genera, and/or families was based on data collected by Gundy and Wurst (1976a, 1976b) and the character matrix built by Lee (1998), in which the presence of a pineal foramen is character number 33. For several species, a parietal foramen was observed in cranial scans collected by Watanabe et al. (2019) and accessible on the Phenome10k website (http://phenome10k.org/), as well as the general scientific literature on anatomy. When data on a particular species were not found, we considered as valid the information on the same genus or family. In general, the presence of a parietal foramen was equaled to the presence of a parietal eye (Edinger 1955). The only exception that we considered was the fossorial, legless Californian Anniellidae lizards, which have a well-developed parietal eye without a corresponding foramen (Gundy and Wurst 1976a, 1976b).

Data on the daily activity patterns of lizards (diurnal, nocturnal, cathemeral) were drawn from the SquamBase database (https://datadryad.org/stash/dataset/doi:10.5061/dryad.76hdr7t3b) compiled by Meiri (2024).

### Phylogenetic Analysis

The evolutionary relationships among OPN1 and OPN3 sequences were performed using the NGPhylogeny program package (Lemoine et al. 2019; https://ngphylogeny.fr/). Protein sequences were aligned with MUSCLE and ambiguously aligned regions and sites were removed with Gblocks (Talavera and Castresana 2007), with the final alignment comprising 249 amino acid positions. Phylogenetic reconstruction was performed using the Maximum Likelihood method as implemented in the PhyML 3.0 program (Guindon et al. 2009, 2010). The model of amino acid substitution, JTT (Jones et al. 1992), was chosen using the SMS program (Lefort et al. 2017) with four categories of substitution rates across sites and estimated gamma parameter, amino acid frequencies, and invariable sites (+G + I + F). Tree refinement was done by the Subtree Pruning and Regrafting (SPR) method, and statistical robustness was evaluated with a fast likelihood ratio test based on the Shimodaira–Hasegawa-like test (SH-like aLRT, Guindon et al. 2009). The final tree was visualized using iTOL (Letunic and Bork 2024; Interactive Tree of Life, https://itol.embl.de/) and IcyTree (Vaughan 2017, https://icytree.org/). All OPNLEP protein sequences analyzed are compiled in supplementary table S5, Supplementary Material online.

## Supplementary Material

evaf058_Supplementary_Data

## Data Availability

All genomes surveyed are available from publicly available databases: Ensembl (http://ensembl.org/index.html/), Ensembl Rapid Release (https://rapid.ensembl.org/index.html/), and the NCBI Genome Database (https://www.ncbi.nlm.nih.gov/datasets/genome/) (supplementary table S4, Supplementary Material online).
