# Supplementary material for: Evolution of Pineal Nonvisual Opsins in Lizards and the Tuatara and Identification of Lepidopsin: A New Opsin Gene
Source: Genome Biol Evol. 2025 May 2;17(5):evaf058. doi: 10.1093/gbe/evaf058 (PMC12043008; doi:10.1093/gbe/evaf058)
Supplement: evaf058_Supplementary_Data [file evaf058_supplementary_data.zip › Romero-deSouza-Suppl Figs.pdf]

```
OPNLEP  ------------MMNHS-------LFSSNALTFLGPNATCNSTTCLDQASEKKYKF-GVG
OPNPT   ------------MENESS-LVLEGAEGYIVRPTIFPR----------------AGYGVLA
OPNPP   -----MDSLNA---------STLSSNASTVRVVLMPR----------------IGYTIIA
OPNP    -----------MWVSNEWINGTPGPFDGPQWPFLAPRGT-----------Y-----TSVA
VA      MSKPDMDGLRREAENDSWLFN-PSSSSAPFDPFLQPLDI-----------IEPWNFHLIS
                                         : *                      :.
                TM1                                  TM2
OPNLEP  VLMILGLFLGFWFNCIIVILTIRHKFLRNPINYAMVNLSVADMFSIVFGLGPTMDANLKG
OPNPT   FLMFINALFSLFNNFLVIAVTLKNPQLRNPINIFILNLSFSDLMMSICGTTIVIATNYHG
OPNPP   IIMATSCTLSVILNTAVIAVTIKYRQLRQPINYSLVNLAIADLGAALLGGSLNVETNAVG
OPNP    VLMGLVMVSAAVVNGLVILVSVQYKRLRSPLNYILVNLAVADLLVTFFGSTISFANNIYG
VA      ALMFVVTLLSLSENFTVILVTIKFKQLRQPLNYVIVNLSVADFLVSLIGGTISFSTNLKG
         :*      .   *  :: ::::   **.*:*  ::**:.:*:   . *    .  *  *
                           TM3                                  TM4
OPNLEP  YSFQSKSFCAFQGFCITLFGLVALWSVVLCAVERYLLVCRPFKALHFGKRHAVLALLFIW
OPNPT   YFYLGRRFCIFQGFAVNYFGIVSLWSLTILAYERYNVVCQPLGTLQMSTQRAYQLLGFIW
OPNPP   YYNLGRVGCVTEGFAMAFFGIVALCTIAVIAVDRAIVIAKPMGTITFTTRKAMIGVAVSW
OPNP    FFVFGPTACEFEGFMVSLTGIVGLWSLAILAFERYLVICKPVGDFRFQQRHAVIGCAFTW
VA      YFYMGHWACVLEGFAVTFFGIVALWSLALLAFERYVVICRPLGNMRLKGKHAALGIAFVW
        :   .   *  :** :   *:*.* ::.: * :*  ::.:*.  : :    *     . *
                                                    TM5
OPNLEP  LWSLIWTLPPLFGWSSYEVEGIASNCALAWHRKDWKSRSYHLSLFFVCFLVPFILMSLSY
OPNPT   VFCLFWAVVPLFGWSSYGPEGVQTSCSIGWEERSWNNYSYLIVYFLSCFFIPVLIIGFSY
OPNPP   IWSLVWNTPPLFGWGGYEMEGVMTSCAPDWANSDPINVSYIICYFLFCFTIPFITILVSY
OPNP    LWSLLWTLPPLFGWSSYIPEGLRTSCGPNWYTGGSDNNSYIMTLFVTCFVTPLAMIVFSY
VA      IFSFIWTVPPIMGWSSYTTSKIGTTCEPNWYSGDYNDHTFIITFFTTCFILPLLVILVSY
        ::.:.*   *::**..*  . : :.*   *   .    :: :  *  **  *.  : .**
                                           TM6
OPNLEP  GMLIFTLRKVAKTGLVQSNSTQRAESQ--VTKTAAIMILAFLLSWMPYAAFALTKVVKPD
OPNPT   GNVIRSLHGLNKK-VEQLGGKSNPEEEFRAVIMVLVMVVAFLICWLPYTLFALTVVFNPA
OPNPP   GYLIWTLRQVAKVGLAQXGSTTKAEAQ--VSRMVIVMVMAFLICWLPYATFALVVVGNPK
OPNP    ANLLLTLRAVAAQ-QKELETTQRAERE--VTRMVITMVMAFLVCWLPYASFAMVVATNKD
VA      GKLMRKLRKVSDT-QGRLGTTRKPERQ--VTRMVVIMILAFLICWSPYAAFSILVTTCPS
        . :: .*: :          . . * :  .   .  *::***:.* **: *::  .
                         TM7
OPNLEP  VTMDPNIQSIPLFMAKTGTIYNPVVYVCLNKQYREQVAASLRCLRKCRVVPSKHRVLNAL
OPNPT   LNISPLAATIPTYLSKTSPVYNPIIYIFLNKEFRECAVEFITCGKVVLTSPEEDISTSAI
OPNPP   IYINPIIATVPMYMAKSSTFYNPIIYIFMNKQFRDCLVRCLLCGRNPCASEQTDEDDLEV
OPNP    LFIQPALASLPSYFSKTATVYNPIIYVFMNKQFRSCLLSTMSCGRRPVAAQGTTPAMISS
VA      IELDPRLAAIPAFFSKTATVYNPVIYVFMNNQFRKCLVQLFRCSDQEIVDANVNPISEKD
        : :.*   ::* :: *:. .***::*: :*:::*.     : *     .

OPNLEP  PPALKRLQGK--QLSCNTNRIAPQNE-----------LNISRNLLLPNMSYSDDESSSQ
OPNPT   SDEGIAPCK--------INQVTPV-----------------------------------
OPNPP   S----------------T--IAPAPPTRRGKVAPI------------------------
OPNP    PRGLTS------ALEGSRNKVVPSASEGSGND--VGTS---------------------
VA      TLAHTKHCGEMSTVAAHVIVFNPRSEDQHGSCQTFAQLAISENKVYPV-----------
                            . *
```

Supplemental Figure S1: Clustal Omega alignment between predicted aminoacid sequences of nonvisual OPN1 opsins from *Anolis carolinensis* (green anole). Predicted transmembrane domains (TM) are highlighted.

Romero & de Souza, 2024

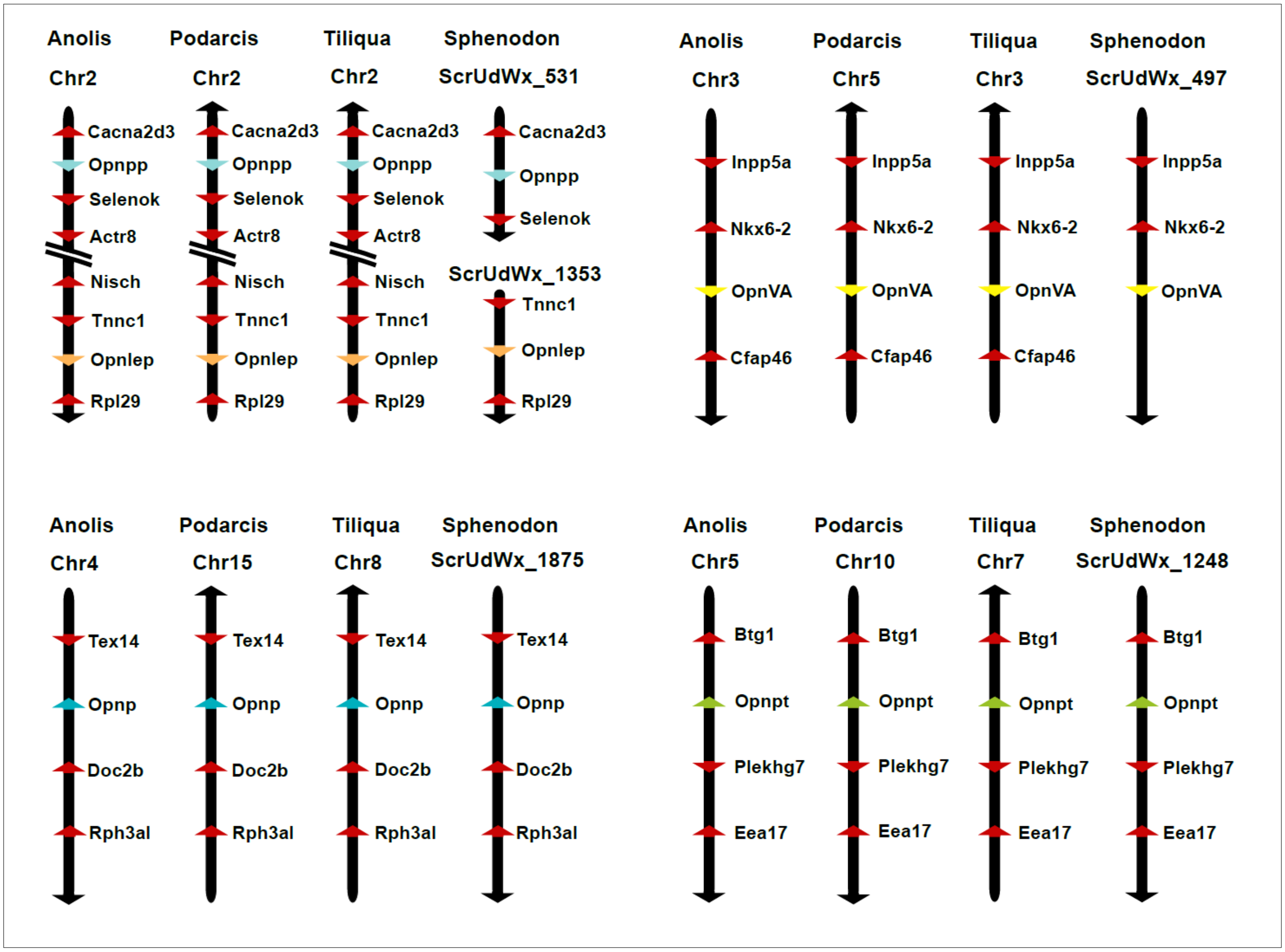

Supplemental Figure S2: Conservation of the chromosomal neighbourhood (synteny) of nonvisual OPN1 genes among Lepidosauria. Species represented are the iguanid *Anolis carolinensis*, the lacertid *Podarcis muralis*, the skink *Tiliqua scincoides* and the tuatara, *Sphenodon punctatus*. Chromosomes are indicated for lizards and unplaced scaffolds for the tuatara.

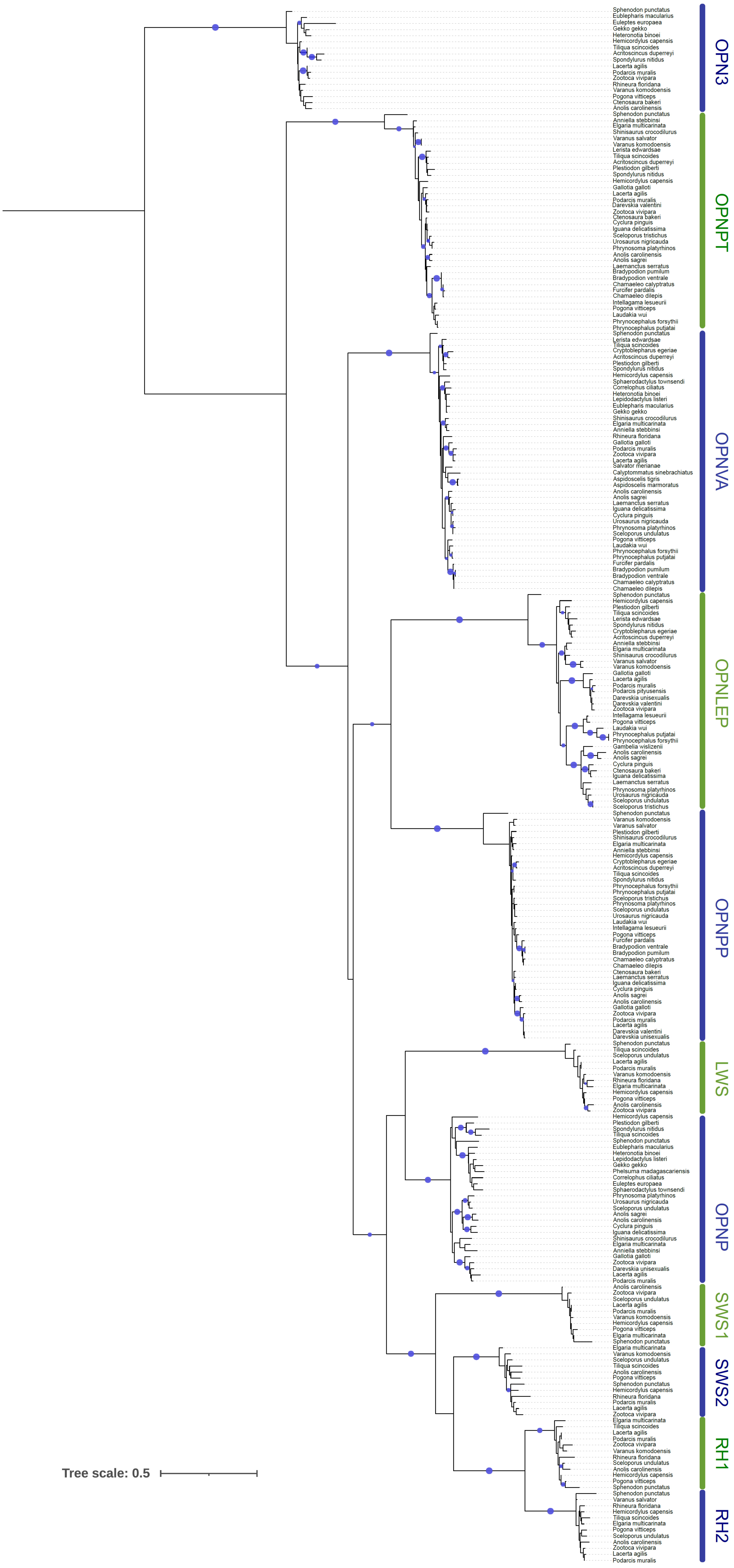

Supplemental Figure S3: Detailed version of the phylogenetic tree shown in Figure 3 with species names and OPN1 and OPN3 opsin clades. Phylogenetic relationships were inferred with the maximum likelihood method implemented in PhyML 3.0 (see Materials and Methods for details). The OPN3 clade was used to root the tree. Blue circles indicate branches with over 0.9 support values (SH-like aLRT test). Note that branches corresponding to each opsin clade, including OPNLEP, are all strongly supported.

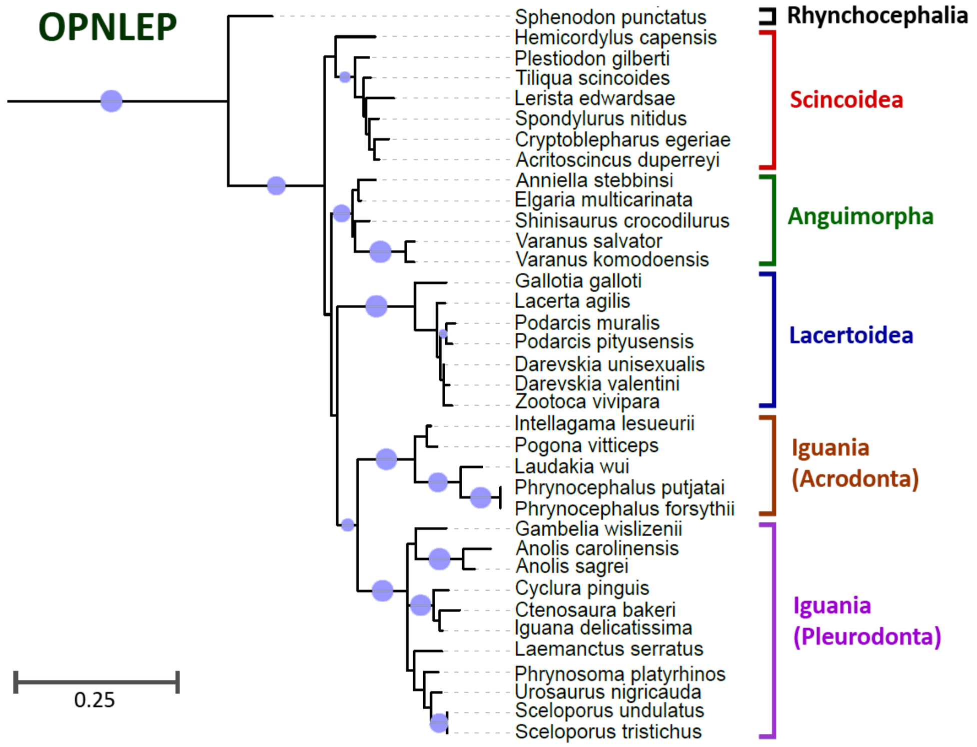

Supplemental Figure S4: Close-up of the OPNLEP branch of the phylogenetic tree shown in Supplemental Figure S3 inferred with PhyML 3.0 (maximum likelihood). OPNLEP proteins are grouped around known lepidosaurian clades. Blue circles indicate branches with >0.9 support values.
